# A non-spectroscopic optical biosensor for the detection of pathogenic *Salmonella Typhimurium* based on a stem-loop DNA probe and retro-reflective signaling

**DOI:** 10.1186/s40580-019-0186-1

**Published:** 2019-05-15

**Authors:** Dong Woo Kim, Hyeong Jin Chun, Jae-Ho Kim, Hyunjin Yoon, Hyun C. Yoon

**Affiliations:** Department of Molecular Science & Technology, Ajou University, Suwon, 16499 Republic of Korea

**Keywords:** *Salmonella*, Molecular diagnostic, Retroreflective Janus particles, Optical biosensor, Stem-loop DNA

## Abstract

The detection of foodborne pathogenic microorganisms is an essential issue in molecular diagnostics. Fluorescence-based assays have been widely utilized in molecular diagnostics because of their ability to detect and measure low analyte concentrations. However, conventional fluorescence-based assays require sophisticated optics systems, such as a specific light source and light filter. To overcome these limitations, we developed an optical sensing system using a retroreflective Janus microparticle (RJP) as a signaling probe. Compared to fluorescent dyes, RJPs have the advantage of not requiring complicated optic systems because they can be observed using visible light without a filter. To confirm that RJPs can be used as a probe for molecular diagnostics, *Salmonella* was detected using a biotinylated stem-loop DNA probe to capture the target gene DNA and a streptavidin-conjugated RJP (SA-RJP) as the detection molecule. When the target gene DNA was present at the sensing surface where the stem-loop DNA probe was immobilized, the biotinylated stem-loop DNA probe was stretched, exposing biotin, which can react with SA-RJP. Since the amount of exposed biotin increased according to the concentration of the applied target gene DNA, the number of observed RJPs on the sensing surface increased with the concentration of the target gene DNA. Consequently, the concentration of *Salmonella* could be quantitated by counting the number of observed RJPs. Using this system, *Salmonella* at concentrations ranging from 0 to 100 nM could be analyzed, with high sensitivity and selectivity, with a limit of detection of 2.48 pM.

**Electronic supplementary material:**

The online version of this article (10.1186/s40580-019-0186-1) contains supplementary material, which is available to authorized users.

## Introduction

*Salmonella* is one of the most common foodborne pathogens. It can infect both animals and humans and cause various symptoms including fever, diarrhea, nausea, vomiting, and even death [1]. According to recent studies, 93.8 million cases of salmonellosis occur every year, resulting in 155,000 deaths annually [2]. Deaths due to *Salmonella* infection occur worldwide, regardless of nationality, including in the United States, the United Kingdom, Australia, and Africa [3]. Therefore, the detection of *Salmonella* is important in the food industry.

Culture-based methods are considered to be standard for the diagnosis of *Salmonella*. However, they are not widely used due to their time-consuming nature. Other disadvantages of these methods include costly and complicated equipment and the need for skilled workers. To overcome these limitations, immunoassay-based colorimetric methods have been introduced as alternatives to culture-based methods for the diagnosis of *Salmonella* [4–7]. Immunoassays based on chromogenic reactions have an advantage that the resulting signals can be quickly and easily detected as a color change. However, these methods require magnetic particles to capture the target pathogen in a pre-enrichment step. To eliminate this pre-enrichment step, direct target-measurement methods have been introduced, including ATP bioluminescence [8], strip-based lateral flow immunoassay [9], surface plasmon resonance (SPR)-based enzymatic precipitation enhancement methods [10], and M13 bacteriophage-based SPR detection methods [11]. Although eliminating the pre-enrichment step allowed *Salmonella* detection in a short time (within 60 min), the limit of detection (LOD) of these assays is relatively high (10^3^–10^4^ CFU/mL).

In recent years, various nucleic acid-based *Salmonella* diagnostic methods have been developed, including fluorescence-based, colorimetric, and electrochemical methods, to improve the sensitivity of detection [12–15]. In particular, methods using stem-loop DNA as a probe has attracted much attention because of the advantage of being able to clearly detect the on–off signal [16–18]. Stem-loop DNA probes consist of two regions; one is the loop region, which has a sequence that is complementary to the target gene DNA, and the other is the stem region, which has a fluorescent molecule at one end and a quencher at the other end. When the target is absent, the stem-loop DNA probe exists as a hairpin structure, and no fluorescence is detected due to the close proximity of the quencher. When the target is present, the stem-loop DNA probe is stretched due to hybridization of the target gene DNA to the loop region, which separates the quencher from the fluorescent molecule, allowing it to fluoresce. These fluorescence-based *Salmonella* detection methods have good sensitivity. Although a lot of effort has been dedicated to the development of such methods for the detection of *Salmonella*, these assays remain challenging because they require a sophisticated instrument.

**Fig. 1:**
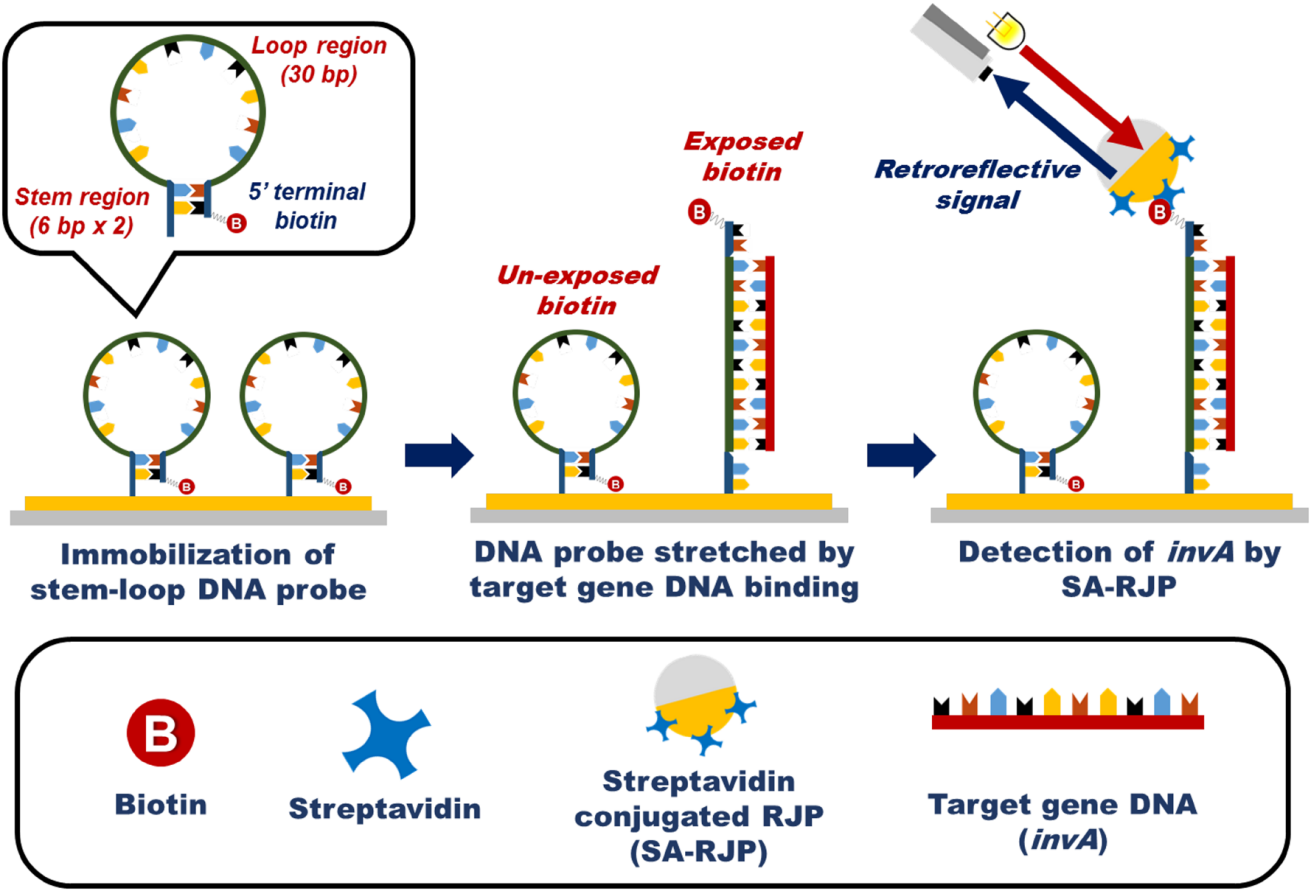
Schematic illustration of the developed *Salmonella* target gene DNA (*invA*) system detection using retroreflective Janus microparticles (RJPs). In the presence of the target gene DNA (*invA*), the stem-loop DNA probe is stretched, exposing biotin. Then, the exposed biotin can be detected using streptavidin-conjugated RJPs. The inset shows the stem-loop DNA probe, which is composed of a loop-region (30 bp), stem-region (6 bp × 2), an amine group at the 3′ end, and biotin at the 5′ end. The sequence of the loop region is complementary to the target gene DNA

In this study, we applied a retroreflective Janus microparticle (RJP) to overcome the requirement for complex equipment in fluorescence-based *Salmonella* diagnosis. An RJP is a non-spectroscopic optical probe that does not require sophisticated equipment for analysis [19–21]. Thus, the integration of an RJP in a stem-loop DNA probe-based *Salmonella* assay will allow highly sensitive detection using a simple optic system. To develop this assay, two DNAs were prepared, the stem-loop DNA probe and target gene DNA. The stem-loop DNA probe, which is modified with biotin, was immobilized on a sensing surface. As shown in the Fig. 1, in the presence of the target gene DNA on the sensing surface, the stem-loop DNA probe would be stretched, exposing biotin. Then, when the streptavidin-conjugated RJP (SA-RJP) is added to the sensing surface, the SA-RJP can react with the exposed biotin. Since the amount of exposed biotin is proportional to the concentration of the target gene DNA, quantitative detection of *Salmonella* can be accomplished by counting the number of observed SA-RJPs. Moreover, this sensing strategy has another advantage as the RJPs can be detected using a simple optical system, such as a white LED and commercial complementary metal-oxide semiconductor (CMOS) camera. The details of the developed sensing system and the experimental results are described below.

## Experimental

### Materials and instruments

A biotinylated stem-loop DNA probe, a target gene DNA, and mismatched target genes were synthesized by Bioneer (Daejeon, Korea). The retroreflective Janus particle-quantifying chips (RQC) were acquired from AMED (Seoul, Korea). Streptavidin and 3,3′-dithiobis (sulfosuccinimidyl propionate) (DTSSP) were obtained from Thermo Scientific (MA, USA). Ethanolamine (EA), tris(hydroxymethyl)aminomethane, sodium dodecyl sulfate (SDS), sucrose, arginine, Tween-20, Triton X-100, polyethylene glycol (PEG), and poly(vinyl alcohol) (PVA) were purchased from Sigma-Aldrich (MO, USA). Bovine serum albumin (BSA) was obtained from BioWorld (OH, USA). Sodium chloride (NaCl) was obtained from Duchefa Biochemie (Haarlem, Netherlands). The storage buffer was purchased from Ademtech (Pessac, France). All other reagents were analytical grade. The VZM™ 450i Zoom Imaging Lens and CMOS Color USB Camera (5 megapixels) were purchased from Edmund Optics (NJ, USA). Reaction buffer solution (RBS) consisted of 10 mM Tris–HCl and 200 mM NaCl, pH 8.0. Washing buffer solution I (WBS I) consisted of 10 mM Tris–HCl, 200 mM NaCl, and 0.1% SDS, pH 8.0. Washing buffer II solution (WBS II) consisted of 20 mM Tris–HCl, 1% BSA, 1% sucrose, 50 mM NaCl, 50 mM arginine, 0.2% Tween-20, 0.2% Triton X-100, 0.2% PEG, and 0.5% PVA, pH 7.4. PBST buffer consisted of 0.02% Triton X-100 in 50 mM phosphate-buffered saline (PBS) solution. All buffer solutions were made with double distilled, deionized water (DDW).

### Preparation of the sensing surface and streptavidin-conjugated RJP

To detect *Salmonella*, a stem-loop DNA was used as a molecular probe, and the sequence is shown in Table 1. The stem-loop DNA probe was immobilized on the RJP-quantifying chip (RQC), which was used as a sensing surface. The real images of RQC (in a microfluidic chip, magnified view, and cross-sectional view) were shown in Additional file 1: Figure S1. Briefly describing the RQC, the RQC contains 16 patterned regions made of gold (340 × 340 μm^2^) and is composed of a 30-nm chromium layer and a 150-nm gold layer on a glass surface, which was generated by using a conventional photolithography technique. The method used to construct the sensing surface was described previously [20]. Briefly, the RQC was cleaned with piranha solution (H_2_SO_4_:H_2_O_2_; 4:1) and then rinsed with DDW three times. Next, DTSSP was applied to the RQC and reacted for 3 h to form the self-assembled monolayer (SAM). Then, the RQC was washed with DDW three times. Next, 20 μL of the stem-loop DNA probe was applied to DTSSP-treated RQC for 1 h, and the amine group on one end of the stem-loop reacted with the sulfo-*N*-hydroxysuccinimide (NHS) ester of DTSSP. During this step, the stem-loop DNA probe was covalently immobilized on the gold surface via an amide bond. To prevent self-hybridization of the stem-loop DNA probes, the reaction temperature was increased to 40 °C. Then, the un-immobilized stem-loop DNA probes were washed away with WBS I (700 μL), and the unreacted NHS moieties of DTSSP were blocked with 20 mM ethanolamine for 30 min.

**Table 1:** The sequences of the stem-loop DNA probe and the target gene

	Sequence
Stem-loop DNA probe	5′-Biotin-(CH_2_)_6_-GTGAGCAAAGAGACCTACCATACGGGCCATTTGTCTGCTCAC-(CH_2_)_6_-NH_2_-3′
Target gene DNA	3′-TTTCTCTGGATGGTATGCCCGGTAAACAGA-5′

In the stem-loop DNA probe, the underlined sequence is the sequence that is complementary to the target gene

Next, the modification of the RJP surface was conducted. The RJP with a diameter of ~ 1.2 μm was synthesized according to the previously reported method [19]. The prepared-RJPs were conjugated to streptavidin, which specifically binds to biotin [20]. In brief, DTSSP (5 mM) was immobilized on the gold region of the RJPs by using the SAM technique for 3 h. After washing away the unreacted DTSSP, streptavidin was conjugated in the same manner used to construct the sensing surface. To block the unreacted region of the RJPs, they were treated with 20 mM ethanolamine for 30 min and with 1% BSA solution for 1 h. Then, the particles were sonicated in the buffer and stored in Ademtech storage buffer until use.

### Optimization of the stem-loop DNA probe concentration

The concentration of the immobilized stem-loop DNA probe was optimized to achieve a high signal-to-noise ratio. For this experiment, various concentrations of the stem-loop DNA probe, ranging from 10 to 50 μM, were immobilized on the RQC. After blocking unreacted DTSSP with 20 mM ethanolamine, 20 μL of the target gene DNA (1 μM) diluted in RBS was applied to the sensing surface where various concentrations of the stem-loop DNA probe were immobilized. To confirm the background signal, 20 μL of RBS containing no target gene DNA was used as a negative control. The measurement procedure was the same as that described in the calibration study in Sect. 2.4.

### Measurement of target gene DNA detected using RJPs

For assessing the molecular diagnosis of *Salmonella* using the developed method, the target gene DNA was injected into the RQC that was modified with the stem-loop DNA probe. The RQC was incubated with the target gene DNA for 45 min at 40 °C, a temperature that is sufficient to allow the stem-loop DNA probe to stretch and expose the biotin. Then, the temperature was reduced to 4 °C for 15 min to promote the formation of the hairpin structure in the stem-loop DNA probes that were not hybridized to the target gene DNA. The unhybridized and non-specifically bound target gene DNAs were washed away with WBS I (700 μL). Then, to minimize non-specific binding between RJPs and the RQC, the sensing surface was blocked with 1% BSA. After blocking for 1 h, the sensing surface was washed with PBST (600 μL). To detect the exposed biotin, SA-RJPs (0.4 mg/mL) were injected into the sensing surface. After 15 min, the unreacted RJPs were washed away with WBS II (700 μL). The RJPs reacted with the biotin were observed with a CMOS camera under a white LED light, and an image of the RJPs was obtained. Then, the number of RJPs in the obtained images was analyzed by counting with NIH Image J.

### Selectivity test with mismatched target genes

To verify the target gene DNA selectivity of the developed RJP-based molecular diagnostic system, single-stranded DNAs with different sequences from that of the target gene DNA were prepared. Mismatched gene 1 (M1), mismatched gene 2 (M2), and mismatched gene 3 (M3) had one, two, and three nucleotide differences, respectively, compared to the target gene DNA sequence. The sequences of the mismatched genes are shown in Table 2. A 20 μL aliquot of each 100 nM mismatched gene diluted in RBS buffer was injected into the prepared sensing surface. For comparison, the same concentration of the target gene DNA and RBS were included as positive and negative controls, respectively. Sample detection was performed as described in Sect. 2.4.

**Table 2:** The sequences of the mismatched genes

	Sequence
Mismatched gene 1 (M1)	3′-TTTCTCTGGATGGTAT**C**CCCGGTAAACAGA-5′
Mismatched gene 2 (M2)	3′-TTTCTCTG**C**ATGGTAT**C**CCCGGTAA ACAGA-5′
Mismatched gene 3 (M3)	3′-TTTCTCTG**C**ATGGTAT**C**CCCGGTA**T**ACAGA-5′

The mismatched bases are shown as bold letters and underlined

## Results and discussion

### Principle of *Salmonella* detection using a retroreflective biosensing platform

*InvA*, which is a gene encoding a protein involved in the invasion of *Salmonella* through a type 3 secretion system, has been widely used as a target gene DNA for the diagnosis of *Salmonella* [22–24]. Therefore, to analyze *Salmonella* with the retroreflective optical detection system developed by us, the *invA* that is conserved in diverse *Salmonella* serotypes was selected as the target gene DNA (427 bp to 456 bp of CDS) [25, 26]. In this study, to detect *Salmonella*, we designed and prepared a stem-loop DNA probe that can hybridize to *invA*. The stem-loop DNA probe, which is capable of specific binding to the target gene DNA, consisted of 42 base pairs. The stem-loop DNA probe has two regions, the loop and stem regions, as well as a biotin residue at the 5′ end and an amine group at the 3′ end with six carbon linkers. The sequences of the stem-loop DNA probe and target gene DNA are shown in Table 1.

The loop region (30 bp) contains the specific sequence that is complementary to *invA*. In addition, the stem region (6 bp × 2), which is present at the 3′ and 5′ ends of the stem-loop DNA probe is capable of forming a double-stranded stem structure. Therefore, in the absence of the target gene DNA, the stem-loop DNA probe exists as a hairpin structure that is formed by hybridization of the two complementary stem regions. The closed form of the stem-loop DNA probe cannot bind to the SA-RJPs because the biotin present at the 5′ end is shielded by steric hindrance [27]. In contrast, in the presence of the target gene DNA, the stem-loop DNA probe is stretched due to hybridization between the target gene DNA and loop region of the stem-loop DNA probe, which exposes biotin. Since the amount of exposed biotin in the stretched stem-loop DNA probe increases in proportion to the increase in target gene DNA concentration, the number of RJPs that can bind to biotin increases proportionally. Therefore, the target gene DNA could be quantitatively analyzed by counting the number of observed RJPs. In addition, because the RJPs can be measured using simple optical equipment, such as a CMOS camera and a white LED, the developed biosensing system has the advantage of being able to detect *Salmonella* without a sophisticated optical instrument.

### Optimization of the concentration of immobilized stem-loop DNA probe

**Fig. 2:**
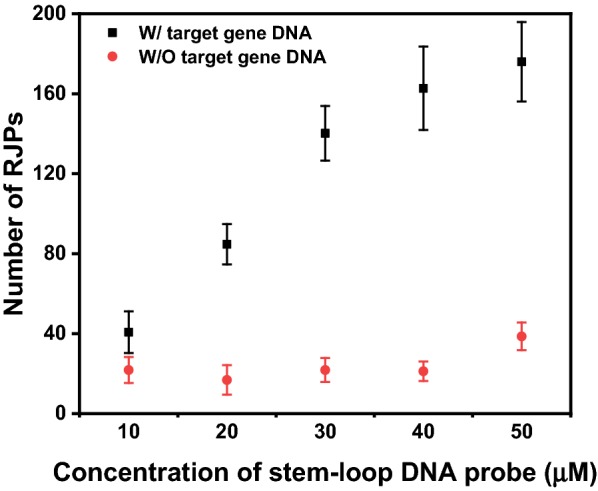
Optimization of the concentration of the stem-loop DNA probe immobilized on the sensing surface. Various concentrations of the stem-loop DNA probe were immobilized to determine the optimal concentration

To effectively detect the target gene DNA, a high signal in the presence of the target gene DNA and a low background signal are required. To achieve this high signal-to-noise ratio, the concentration of the immobilized stem-loop DNA probe should be optimized. Prior to determining the optimal concentration of the stem-loop DNA probe, since the RJP and RQC were used as an optical probe and sensing surface, respectively, it was necessary to confirm that the DNA and streptavidin were immobilized on the RQC and RJP. In a previous study, we confirmed the streptavidin modification on the RJP surface and that the stem-loop DNA probe was immobilized on the RQC [20]. Therefore, we assumed that the RJP and RQC could be used in this study as an optical probe and sensing surface, respectively, without verification. To determine the optimal concentration of the stem-loop DNA probe, various concentrations of the probe (10–50 μM) were applied to the RQC, and the number of observed RJPs was measured (Fig. 2).

As shown in Fig. 2, in the absence of the target gene DNA, the numbers of observed RJPs were similar regardless of the concentration of the stem-loop DNA probe immobilized on the sensing surface, because the biotin was not exposed. These values were considered to be the background signal, indicating that the developed biosensing surface has a constant background signal.

In contrast, in the presence of 1 μM target gene DNA, the number of observed RJPs increased in proportion to the concentration of the immobilized stem-loop DNA probe. As shown in Fig. 2, ~ 162.7 RJPs were observed when the stem-loop DNA probe concentration was 40 μM. Similarly, about 176.0 RJPs were observed in the presence of 50 μM stem-loop DNA probe. However, the background signal at the 50 μM stem-loop DNA probe concentration was 38.6 RJPs, which is relatively higher than the signal observed at 40 μM (21.2 RJPs). Although the observed signal was the highest at 50 µM stem-loop DNA probe, it was not significantly higher than the signal at 40 μM. Furthermore, a low background signal was measured at 40 μM. These results indicate that 40 μM was sufficient to detect the target gene DNA with a high signal-to-noise ratio. Therefore, the optimal stem-loop DNA probe concentration was 40 μM.

### Calibration study for the developed retroreflective biosensing platform using a stem-loop DNA probe and RJPs

To confirm that the developed stem-loop DNA-based molecular biosensing system is capable of detecting *Salmonella*, a diagnostic test was performed. Since the purpose of this study was to determine if quantitative analysis of *Salmonella* is possible, we prepared and analyzed various concentrations of the target gene DNA (0.01–100 nM). Each target gene DNA solution was injected onto an RQC with immobilized stem-loop DNA probe (at 40 μM), and the RQCs were incubated at a temperature above the T_m_ of the stem structure in the stem-loop DNA probe. During incubation, the stem-loop DNA probes are unfolded, allowing hybridization between the loop region and the target gene DNA, and thus exposing the biotin at 5′-terminal. Then, to refold the hairpin structure of stem-loop DNA probes that did not bind to the target gene DNA, the RQCs were incubated at the temperature of 4 °C for 15 min. Then, the SA-RJPs were injected onto the RQCs. As described in principle section, the SA-RJPs could react with the biotin present at the end of a stretched-stem-loop DNA probe. Since the number of reacted RJPs is positively correlated with the concentration of the target gene DNA, quantitative analysis of the target gene DNA could be achieved by counting the number of observed RJPs.

**Fig. 3:**
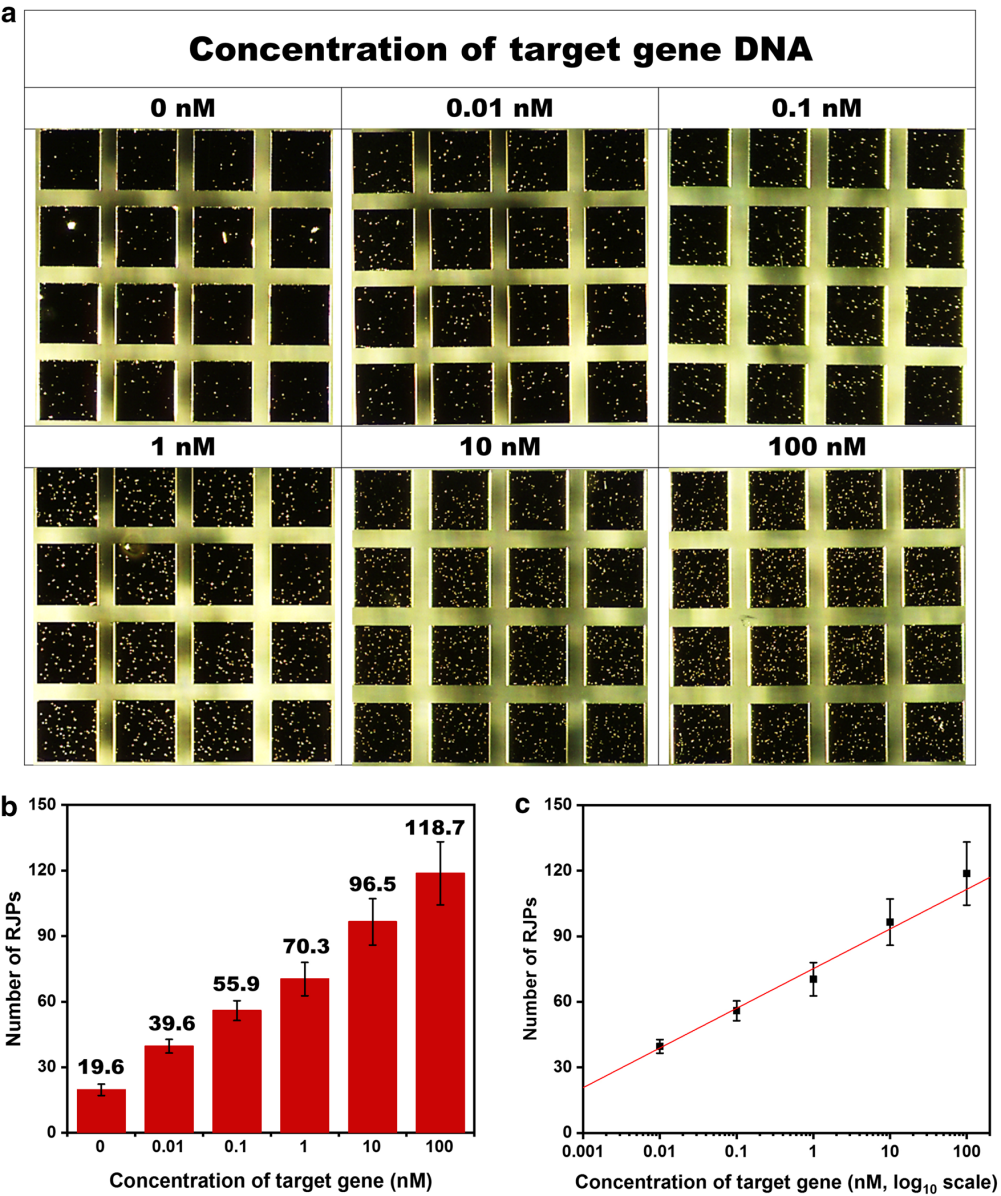
**a** Images of the RJPs on the RQC in a *Salmonella* detection assay with various *invA* concentrations (0, 0.01, 0.1, 1, 10, and 100 nM) performed using the developed optical sensing platform. **b** The graph shows the number of bound RJPs, which was calculated from images using Image J. **c** The calibration curve for the *invA* assay on a log scale. Linear regression analysis was performed, and the error bars are the standard deviation

Figure 3a shows an image of results acquired with the CMOS camera. The number of RJPs in the image increased as the concentration of target gene DNA increased. For the quantitative analysis, NIH Image J was used to count the number of RJPs in the image. The number of counted RJPs is shown in Fig. 3b. When the target gene DNA concentration was 0 nM, ~ 19.6 RJPs were detected. This value could be regarded as the background signal due to non-specific binding of the SA-RJPs to the RQC and because the target gene DNA is not present. To verify the reproducibility of the background signal, the same experiment was carried out at least five times, and the standard deviation of the mean was ~ 10%. The background signal in the calibration study was similar to the background signal shown in Sect. 3.2, indicating that the obtained background signal is reliable. When the concentration of the target gene DNA was 100 nM, 118.7 RJPs were detected. As the concentration of the target gene DNA increased, the number of observed RJPs increased by 20 RJPs for each tenfold increase from 0.01 to 100 nM. Figure 3c shows a linear calibration graph that was obtained from experiments using 0–100 nM target gene DNA. Using the acquired calibration curve, the limit of blank (LOB) and limit of detection (LOD) of the developed biosensing system were calculated as 1.47 pM and 2.84 pM, respectively. A number of studies have been conducted regarding the *Salmonella* detection by using fluorescence-based detection. Reported LOD values from these papers were around 1 nM or 2 CFU (colony forming unit), showing the competitiveness of current approach [16, 28, 29]. In addition, while the fluorescence-based sensing requires sophisticated optical activation/detection setup, the retroreflective non-spectroscopic sensing could be conducted with the simple optical system.

### Verification of the selectivity of the developed molecular diagnosis system

To be a useful biosensor capable of detecting and measuring *Salmonella* contamination, the RJP-based optical signals obtained with the developed biosensing system should originate from DNA–DNA interaction between the stem-loop DNA probe and the target gene DNA. In other words, the stem-loop DNA probe should be unfolded only in the presence of the target gene DNA. Therefore, to confirm that the stem-loop DNA probe is not unfolded by the presence of other genes with similar sequences, a selectivity test was performed. For the selectivity test, single-strand DNAs that differ from the target gene DNA by several nucleotides were prepared (Table 2).

These genes were named mismatched gene 1 (M1), mismatched gene 2 (M2), and mismatched gene 3 (M3) depending on the number of different nucleotides (the bold red underlined bases in Table 2 are different from the target gene DNA). When these mismatched genes were injected onto the stem-loop DNA probe-immobilized RQC, the mismatched genes did not perfectly hybridize to the stem-loop DNA probe, and the stem-loop DNA probe did not stretch, unlike when the target gene DNA, which has a complementary sequence, was hybridized to the stem-loop DNA probe. Therefore, the number of detected RJPs was fewer than when the complementary target gene DNA was used, because fewer biotin molecules are exposed than in the presence of the target gene DNA.

**Fig. 4:**
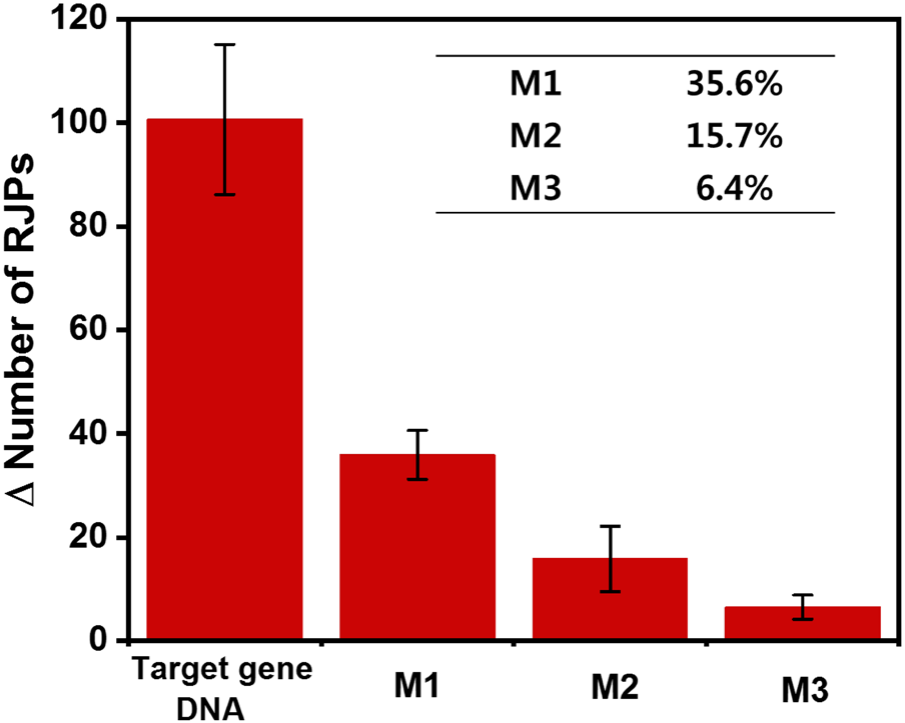
Results of the selectivity test. The change (Δ) in the number of RJPs detected was calculated by subtracting the background from the number of RJPs detected. The target gene DNA, mismatched gene 1 (M1), mismatched gene 2 (M2), and mismatched gene 3 (M3) were used at a concentration of 100 nM. The inset shows the ratio of RJPs for the mismatched genes compared to the target gene DNA, which was set to 100%

Figure 4 shows the number of observed RJPs when these mismatched genes (diluted in RBS to 100 nM) were injected onto the developed biosensing system. For a more accurate comparison with the results obtained using the target gene DNA, the change in the number of RJPs was used, which was obtained by subtracting the background signal from the number of observed particles. For M1, the change in the number of observed RJPs was 35.9, which was 35.6% of the number of RJPs detected with the target gene DNA. Similarly, when M2 and M3 were tested with the developed biosensing system, the change in the number of observed RJPs was 15.8 and 6.4, which was calculated to be 15.7% and 6.4% of that for the target gene DNA, respectively. In previous studies, when one mismatched nucleotide was present in a sequence, ~ 30% of the signal obtained with the complementary target gene DNA was detected [30–32]. Since the results obtained from the developed biosensing system show the same tendency as that reported previously, the selectivity of the developed biosensing system is believed to be reliable. Taken together, these results show that the developed biosensing system has excellent selective detection capability for *Salmonella*.

## Conclusions

In this study, we developed a *Salmonella* sensing platform using RJPs with a simple optical system. The developed sensing platform using RJPs does not require sophisticated optical equipment, compared with conventional fluorescence-based detection systems for *Salmonella*, because the RJPs can be observed using a common white LED and a CMOS camera. Using this sensing system, highly sensitive, quantitative analysis of *Salmonella* was successfully achieved. In addition, we confirmed that the developed system has high selectivity for *invA* using oligonucleotides with mismatched sequences. Based on the results of this research, we believe that the developed sensing system is an attractive platform for the detection of food-borne pathogens such as *Salmonella*.

## Additional file

**Additional file 1: Figure S1.** (A) The image of RQC (RJP-quantifying chip); (B) The magnified view of the sensing surface of RQC; (C) Schematic illustration of the cross-section of RQC.

## Abbreviations

RJP: retroreflective Janus microparticle
SA-RJP: streptavidin-conjugated RJP
SPR: surface plasmon resonance
LOD: limit of detection
CFU: colony forming unit
CMOS: commercial complementary metal-oxide semiconductor
LED: light emitting diode
RQC: retroreflective Janus particle-quantifying chips
DTSSP: 3,3′-dithiobis (sulfosuccinimidyl propionate)
SAM: self-assembled monolayer
WBS: washing buffer solution
RBS: reaction buffer solution
NHS: sulfo-*N*-hydroxysuccinimide
EA: ethanolamine
SDS: sodium dodecyl sulfate
PEG: polyethylene glycol
PVA: poly(vinyl alcohol)
BSA: bovine serum albumin
NaCl: sodium chloride
DNA: deoxyribonucleic acid
PBS: phosphate buffer saline
PDMS: polydimethylsiloxane
