# Supplementary material for: A non-spectroscopic optical biosensor for the detection of pathogenic Salmonella Typhimurium based on a stem-loop DNA probe and retro-reflective signaling
Source: Nano Converg. 2019 May 15;6:16. doi: 10.1186/s40580-019-0186-1 (PMC6517456; doi:10.1186/s40580-019-0186-1)
Supplement: Supplementary file 1 — Additional file 1: Figure S1. (A) The image of RQC (RJP-quantifying chip); (B) The magnified view of the sensing surface of RQC; (C) Schematic illustration of the cross-section of RQC. [file 40580_2019_186_MOESM1_ESM.docx]

***Additional information***

A non-spectroscopic optical biosensor for the detection of pathogenic *Salmonella* Typhimurium based on a stem-loop DNA probe and retro-reflective signaling

Dong Woo Kim, Hyeong Jin Chun, Jae-Ho Kim, Hyunjin Yoon, Hyun C. Yoon*

*Department of Molecular Science & Technology, Ajou University, Suwon 16499, Republic of Korea*

* Author to whom correspondence should be addressed

E-mail: hcyoon@ajou.ac.kr

Tel: 82-31-219-2512

**Figure S1**


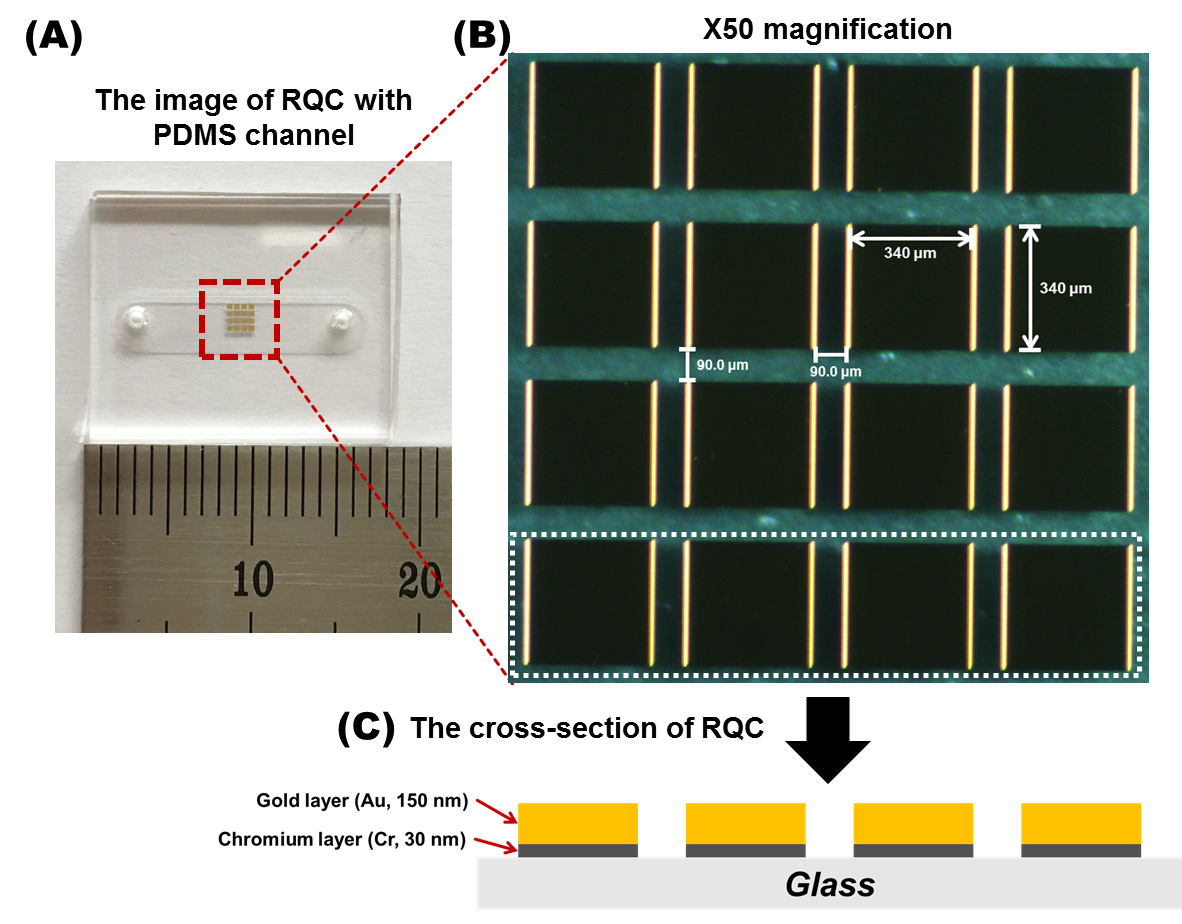


**Figure S1.** (A) The image of RJP-quantifying chip (RQC). (B) The magnified view of the sensing surface of RQC. (C) Schematic illustration of the cross-section of RQC having chromium layer (30 nm) and gold layer (150 nm).
